# Preliminary Evidence on Safety and Clinical Efficacy of Luteolin for Patients With Prostate Cancer Under Active Surveillance

**DOI:** 10.1155/proc/8165686

**Published:** 2025-02-25

**Authors:** Taku Naiki, Aya Naiki-Ito, Akihiro Murakami, Hiroyuki Kato, Yosuke Sugiyama, Tatsuya Kawai, Shinji Kato, Toshiki Etani, Takashi Nagai, Nobuhiko Shimizu, Toshiharu Morikawa, Maria Aoki, Masakazu Gonda, Xiaochen Kuang, Yuko Nagayasu, Shuzo Hamamoto, Takahiro Yasui, Satoru Takahashi

**Affiliations:** ^1^Department of Experimental Pathology and Tumor Biology, Nagoya City University Graduate School of Medical Sciences, Nagoya, Japan; ^2^Department of Nephro-urology, Nagoya City University Graduate School of Medical Sciences, Nagoya, Japan; ^3^Department of Radiology, Nagoya City University Graduate School of Medical Sciences, Nagoya, Japan

**Keywords:** AR, luteolin, miRNA-29, miRNA-30, prostate cancer

## Abstract

**Background:** A need exists for effective treatments for prostate cancer (PCA) due its re-emergence following androgen deprivation therapy, a major clinical problem. In a previous study, we presented evidence on the chemopreventive and chemotherapeutic potential of luteolin, a flavonoid, in PCA including castration-resistant PCA. In this single-arm phase I study, we clinically examined the safety of the oral intake of luteolin in patients under active surveillance (AS).

**Methods:** Between March and September in 2022, five patients with low–intermediate risk PCA and under AS were treated daily with 50 mg of oral luteolin for six months. We investigated the efficacy of oral luteolin in oncological outcomes and any adverse events (AEs) and examined prostate and blood specimens.

**Results:** The median age of patients was 68 years (range: 60–78), and the median initial prostate-specific antigen level was 9.5 ng/mL. All patients were under AS without rapid progression. After treatment with luteolin, AEs were not noted in any patients for six months. All patients underwent a protocol biopsy. Of these, two patients showed a favorable response, one patient had stable disease, and two patients showed disease progression; robot-assisted radical surgery was subsequently performed for the latter. Immunohistochemical analysis revealed decreased expression of androgen receptor and NKX3.1 in noncancerous lesions after luteolin treatment. In addition, quantitative reverse transcription-PCR revealed that serum micro(mi)RNA expression in serum and prostate gland, including miR-29 and miR-30, tended to be upregulated after luteolin treatment compared with during the pretreatment phase.

**Conclusions:** Our small phase I study of men with PCA suggests that daily treatment with 50 mg of an oral supplement of luteolin is safe and effective with regard to oncological outcomes, particularly in patients under AS.

## 1. Introduction

In the United States, prostate cancer (PCA) is the most commonly diagnosed cancer in men. An estimated 288,300 new cases of PCA will be diagnosed in 2023, with the majority of patients newly diagnosed with localized disease [1]. As a strategy, active surveillance (AS) is aimed at avoiding overtreating those patients that have a lower risk of localized PCA. Currently endorsed by a number of professional societies, AS has increasingly been adopted as a standard of care for such patients [2–6]. Local treatments, such as radiation therapy, brachytherapy, external beam radiation therapy, or radical prostatectomy, can cause adverse events (AEs) including sexual and voiding dysfunctions. Therefore, based on the accumulation of data on AS [7], treatment guidelines of the National Comprehensive Cancer Network recommend this for the management of low–intermediate risk PCA (https://www.nccn.org/). However, in approximately 20%–50% of patients, AS was related to conversion to localized therapy. Several articles prospectively assessed the value of medical interventions to reduce progression rates from AS after commercially available, approved hormonal drugs were developed. However, additional interventional approaches to reduce the risk of disease progression and as new treatment strategies without AEs are strongly desired.

Previous data suggested that dietary foods deficient in polyphenol or other natural plant-based ingredients, including herbs, teas, and spices, have been linked to a higher risk of developing PCA [8, 9]. In addition, patients adopting healthy diets after being diagnosed with PCA were shown to have slower lethal PCA progression [10, 11]. In such foods, the major components include carotenoids, vitamin E, and polyphenols, including flavonoids. These have antioxidant properties that are thought to protect cellular DNA against oxidative damage from environmental carcinogens [12, 13]. Compared with several other flavonoids or antioxidants, luteolin (3′, 4′, 5, 7-tetrahydroxyflavone) has anti-inflammatory and antioxidative properties. It is present in celery, perilla seeds, parsley, and green pepper [14] and acts as an anticancer agent in cancers of the colon, breast, and liver, among others [15]. Luteolin induces cellular cell-cycle arrest and apoptosis and downregulates the insulin-like growth factor-1 receptor (IGF-1R) signaling pathway [16]. In a previous study, we newly established a castration-resistant PCA animal model [17–20] in which an antioxidative stress pathway was highly induced under castration conditions [21–23]. In addition, we showed that luteolin is a strong suppressive therapeutic agent via the downregulation of androgen receptor (AR), NKX3.1 (a transcriptional factor regulated by AR), and IGF-1R protein expression in a castration-resistant PCA cell line, 22Rv1 [24]. Moreover, we showed that by microarray analysis, several suppressive micro(mi)RNAs, including miRNA-29, and miRNA-30, were upregulated by luteolin treatment [24]. More recently, low-dose luteolin has become clinically available as supplemental medicine for lowering uric acid. However, data do not exist as to the clinical efficacy of oral luteolin in patients with cancer.

Therefore, in this study, we performed a safety analysis for a dispensed supplement of 50 mg of luteolin for patients with PCA under AS in a small phase I study.

## 2. Materials and Methods

### 2.1. Patient Enrollment

We undertook a single-arm, phase I study of a daily dispensed supplement of 50 mg luteolin for 180 days in the absence of castration in men under AS. All patients had low–intermediate risk, localized PCA diagnosed by prostate biopsy (with or without multiparametric magnetic resonance imaging [mpMRI]–guidance) and were recruited six months after diagnosis. The enrollment criteria for patients were as follows: (i) clinical stage cT1c or cT2a disease; (ii) initial prostate-specific antigen (PSA) levels < 15 ng/mL; (iii) grade group (GG) 2 present in ≤ 50% of two cores/site; and (iv) GG1 disease in all other cores. At 1, 3, and 6 months after the initiation of luteolin treatment, the patients underwent a blood examination for hematological parameters and serum chemistries, including blood cell counts, hepatic and renal function, electrolyte composition, testosterone, and PSA, and an electrocardiogram. In addition, AEs were based on National Cancer Institute Common Terminology Criteria for AEs (CTCAE), v. 4.0.

Following 180 days of therapy, all patients underwent a repeated protocol prostate biopsy after an mpMRI. For the repeated protocol biopsy, when a suspicious lesion was observed in the mpMRI, patients underwent prostate biopsies targeting sites (two cores from each positive mpMRI lesion) according to a transrectal ultrasound (TRUS)/mpMRI-guided fusion protocol and following a 12-core systematic biopsy using a BioJet software system (D&K Technologies GmbH, Barum, Germany) as previously described [25]. When a suspicious lesion was not observed with mpMRI, only 12-core systematic biopsies guided by TRUS were performed. In the repeated protocol prostate biopsy, an increase in positive cores or a worsening of the GG was defined as progression. In this case, radical treatment, including radical prostatectomy or radiation therapy, was recommended. Alternatively, all patients continued under AS in outpatient clinics.

The primary endpoint was safety following 180 days of supplemental luteolin treatment. Secondary endpoints included a negative repeat biopsy at the protocol biopsy 180 days after treatment and a change in immunohistochemical profiles in a biopsy specimen before and after luteolin treatment. In addition, the serum miRNA status before and after luteolin treatment was also analyzed.

### 2.2. Immunohistochemistry

Formalin-fixed paraffin-embedded (FFPE) PCA samples were sectioned and incubated with antibodies against AR (Sigma-Aldrich Corporation, St Louis, MO, USA), NKX3.1 (Cell Signaling Technology, Danvers, MA, USA), and Ki67 (Agilent Technologies, Santa Clara, CA, USA) [26]. The labeling indices of each stain were scanned using an image analyzer and BZ-9000 fluorescence microscope (Keyence, Osaka, Japan) and quantified using software (BZ-analysis application, Keyence).

### 2.3. RNA Extraction and Quantitative Reverse Transcription (qRT)-PCR for miRNA

For qRT-PCR, total RNA from serum was isolated using an miRNeasy Serum/Plasma Advanced Kit (Qiagen, Hilden, Germany). Prostate gland–derived RNA was extracted from biopsied FFPE specimens. Two 10-μm-thick slices of the prostate gland FFPE block were extracted using a miRNeasy FFPE Kit (Qiagen). A 5-ng sample of RNA was converted to cDNA (TaqMan Advanced miRNA cDNA Synthesis Kit, Thermo Fisher Scientific, Rockford, IL, USA). Relative miRNA expression was measured using TaqMan Advanced miRNA Assays (Thermo Fisher Scientific) in an AriaMx Real-Time PCR System (Agilent Technologies). Expression analysis in miRNA was performed by the relative quantification method (ΔCt method). miR-16-5p has been reported to be stably expressed in tissue [27] and serum [28] and was used as a normalizer in this study.

### 2.4. Statistical Analysis

The concentration levels of lactate dehydrogenase, as well as changes from baseline in other variables, including aspartate transaminase, alanine aminotransferase, albumin (Alb), total protein, creatinine (Cre), blood urea nitrogen, testosterone, PSA, and PSA density, were assessed. Descriptive statistics including mean, standard deviation, percentage, and frequency were used for data analysis, and repeated measures analysis of variance (ANOVA) was used to compare the difference between median scores of baseline (pretreatment) and 1, 3, and 6 months after intervention. Mauchly's test was employed to assess the hypothesis of sphericity. If this assumption was violated, analyses were based on the Greenhouse–Geisser test. Peer-to-peer comparisons were performed using a Bonferroni post hoc test. A *p* value < 0.05 was considered significant.

## 3. Results

### 3.1. Safety Analysis of Oral Luteolin

**Figure 1:**
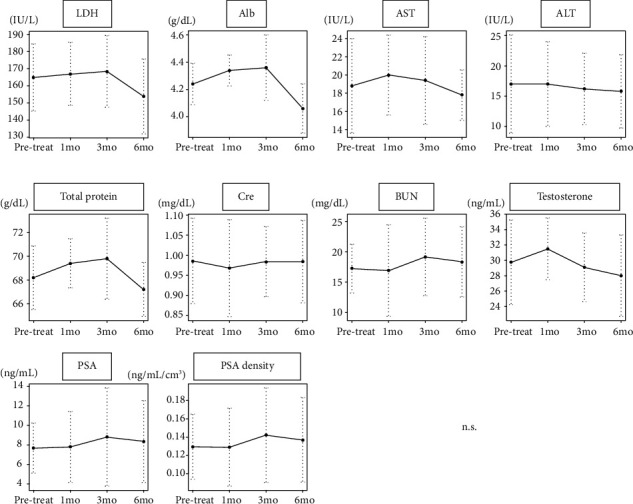
Profiles of AEs via the daily oral intake of 50 mg luteolin for 24 weeks. AEs, adverse events; Alb, albumin; ALT, alanine aminotransferase; AST, aspartate transaminase; BUN, blood urea nitrogen; Cre, creatinine; LDH, lactate dehydrogenase; n.s., not significant; PSA, prostate-specific antigen.

Baseline disease characteristics are listed in Table 1. In this study, a total of five patients (three low risk and two intermediate risk) were enrolled. All patients completed treatment of 50 mg oral luteolin for 180 days without any deficiency. As shown in Figure 1, in a blood examination, no significant change was noted in hematological parameters and serum chemistries 1, 3, and 6 months after treatment. Consecutive PSA and PSA density levels also showed no statistical significance during the administration periods. In spite of detailed interviews, symptomatic AEs evaluable by CTCAE were not found. Of the five patients, two showed a negative prostate biopsy (favorable response: FR), one patient had the same positive cores (stable disease: SD), and two patients showed worsening GG or increasing positive cores (progressive disease: PD) in a repeated protocol prostate biopsy.

**Figure 2:**
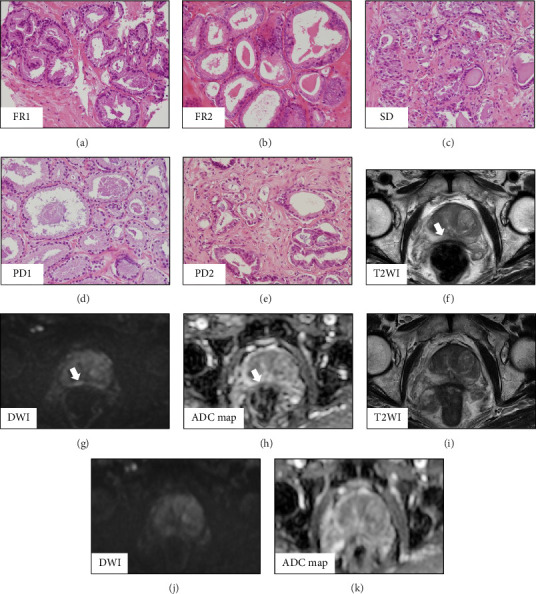
Pathological findings of cancerous lesions at initial diagnosis and consecutive MRI depictions in an FR1 patient. An FR1 patient had two cores of GG1 (a); an FR2 patient had one core of GG1 (b); an SD patient had two cores of GG2 (c); a PD1 patient had one core of GG1 (d); and a PD2 patient had two cores of GG1 (e) at diagnosis. Of these, the FR1 patient showed a positive lesion by mpMRI at diagnosis (f–h) and by TRUS/mpMRI fusion biopsy. Two cores of a GG1 cancer specimen were obtained from an mpMRI-targeted lesion at the initial prostate biopsy. After 180 days of luteolin treatment, the positive lesion had completely disappeared (i–k). ADC, apparent diffusion coefficient; DWI, diffusion weighted images; FR, favorable response; GG, grade group; mpMRI, multiparametric magnetic resonance imaging; PD, progressive disease; SD, stable disease; T2WI, T2-weighted images; TRUS, transrectal ultrasound. Arrow: targeted cancer lesion.

In pathological findings of cancerous lesions, Figure 2 shows the cancer cores for each patient: FR1 patient had two cores of GG1 (Figure 2(a)); FR2 patient had one core of GG1 (Figure 2(b)); SD patient had two cores of GG2 (Figure 2(c)); PD1 patient had one core of GG1 (Figure 2(d)); and PD2 patient had two cores of GG1 (Figure 2(e)) at diagnosis. Of these, the FR1 patient showed a positive lesion in mpMRI at diagnosis (Figures 2(f), 2(g), and 2(h)); by TRUS/mpMRI fusion biopsy, two cores of a GG1 cancer specimen were obtained from an mpMRI-targeted lesion at the initial prostate biopsy. And after 180 days of daily luteolin treatment, the positive lesion had completely disappeared (Figures 2(i), 2(j), and 2(k)). At a repeated protocol biopsy for the FR1 patient, for a precise estimation of the effectiveness of luteolin, in addition to the systematic 12-core biopsy, two cores of TRUS/mpMRI-targeted fusion biopsies based on mpMRI mapping at diagnosis were also added; however, no cancer cores existed in the protocol biopsy specimen.

Regarding the PD patients, one patient was 67 years old. Their initial PSA level was 11.8 ng/mL with a core of GG1 adenocarcinoma in a prostate biopsy at diagnosis and classified into intermediate as D'Amico risk category. After luteolin treatment, this proved to be a GG3 adenocarcinoma in a protocol biopsy at a 15.3 ng/mL PSA level. Another patient was 61 years old. Their initial PSA was 7.2 ng/mL with a core of GG1 adenocarcinoma in a biopsy at diagnosis and classified into low as D'Amico risk category. After luteolin treatment, this proved to be a GG2 adenocarcinoma in a protocol biopsy at a 7.6 ng/mL PSA level. Under a diagnosis of progression, they underwent robotic radical surgery with no signs of PSA recurrences occurring in either patient.

### 3.2. Biological Analysis of Luteolin Treatment Using Consecutive Blood Samples and Specimens in a Repeated Protocol Biopsy

**Figure 3:**
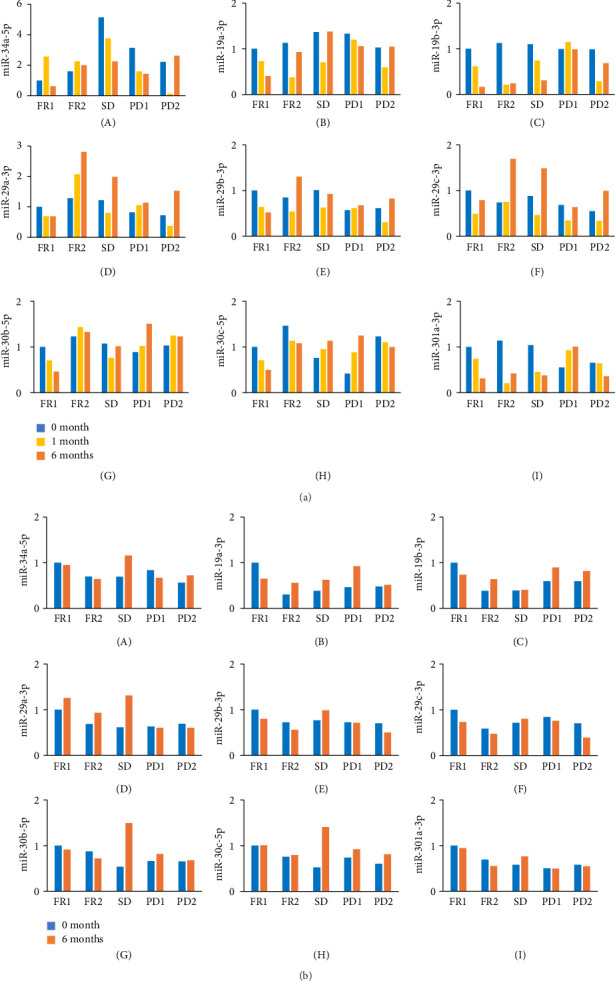
(a) Consecutive miRNA expression levels in sera of all five patients: (A) miR-34a-5p, (B) miR-19a-3p, (C) miR-19b-3p, (D) miR-29a-3p, (E) miR-29b-3p, (F) miR-29c-3p, (G) miR-30b-5p, (H) miR-30c-5p, and (I) miR-301a-3p. FR, favorable response; miRNA, microRNA; PD, progressive disease; SD, stable disease. (b) Expression levels of miRNA before and after luteolin treatment in prostate gland of all five patients: (A) miR-34a-5p, (B) miR-19a-3p, (C) miR-19b-3p, (D) miR-29a-3p, (E) miR-29b-3p, (F) miR-29c-3p, (G) miR-30b-5p, (H) miR-30c-5p, and (I) miR-301a-3p. miRNA, microRNA.

To determine the effects of luteolin, qRT-PCR analysis of miRNAs in consecutive serum and prostate samples was performed. As shown in Figures 3(a) and 3(b), although it varied depending on the case, levels of miR-29a-3p, miR-29b-3p, and miR-29c-3p in serum and miR-29a-3p, miR-30b-5p, and miR-30c-5p in prostate gland particularly in SD patient tended to increase after 6 months of luteolin treatment compared with pretreatment levels (Figures 3(a)(D), 3(a)(e), 3(a)(F), 3(b)(D), 3(b)(G), and 3(b)(H)).

**Figure 4:**
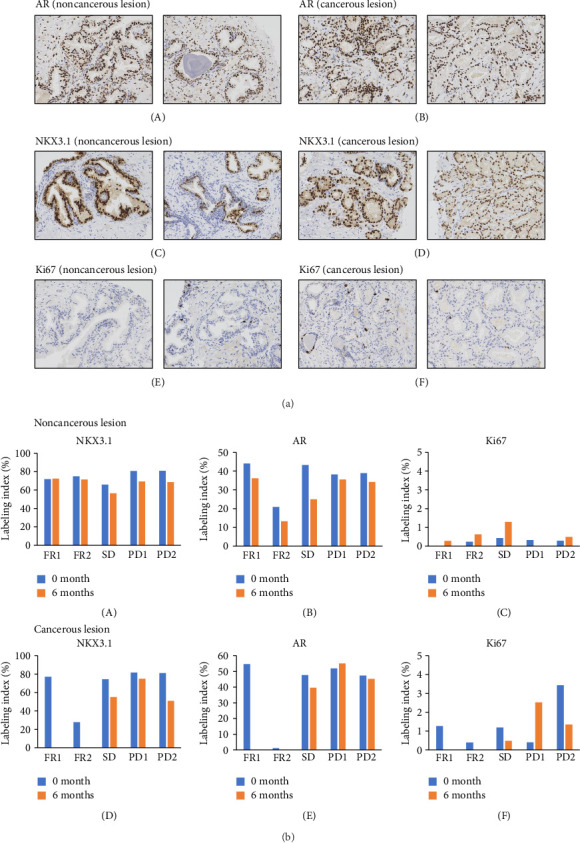
(a) Representative immunohistochemical stains. (A) AR in a noncancerous lesion. (B) AR in a cancerous lesion. (C) NKX 3.1 in a noncancerous lesion. (D) NKX 3.1 in a cancerous lesion. (E) Ki67 in a noncancerous lesion. (F) Ki67 in a cancerous lesion. AR: androgen receptor. (b) Quantification of expression in immunohistochemical analyses. (A) NKX3.1 in a noncancerous lesion. (B) AR in a noncancerous lesion. (C) Ki67 in a noncancerous lesion. (D) NKX3.1 in a cancerous lesion. (E) AR in a cancerous lesion. (F) Ki67 in a cancerous lesion. AR, androgen receptor; FR, favorable response; PD, progressive disease; SD, stable disease.

Immunohistochemical images of the specimens obtained by repeated protocol biopsy are shown in Figure 4(a). It was found that in noncancerous lesions, NKX3.1 and AR expression tended to decrease after 6 months of luteolin treatment compared with pretreatment expression (Figures 4(b)(A) and 4(b)(B)) though Ki67 expression levels were different depending on the case (Figure 4(b)(C)). Also, in cancerous lesions, NKX3.1 and AR expression tended to decrease after luteolin treatment (Figures 4(b)(D) and 4(b)(E)). Ki67 expression in the SD patient tended to decrease after treatment (Figure 4(b)(F)).

## 4. Discussion

PCA re-emerging following androgen deprivation therapy is a major clinical problem. AS aims to avoid the overtreatment of patients with a lower risk of localized PCA. For such patients, a healthy diet that included flavonoids such as luteolin, with its anti-inflammatory and antioxidative properties, was shown to slow lethal PCA progression [10, 11].

In a phase I study of five patients with PCA, we found that 180 days of 50 mg luteolin treatment was generally well-tolerated. In addition, all five patients did not experience AEs over time. The dispensed supplement of luteolin used in this study was originally prepared as supplemental medicine to lower uric acid. At this concentration, prior reports on long-term treatments in people, including patients with cancer, are lacking.

For intervention in patients with low–intermediate risk PCA under AS, several phase II studies described using commercially available and approved hormonal drugs for a benign prostate (BPH) or antiandrogen agents or gonadotropin-releasing hormone agonists for the attenuation of PCA progression [29–33]. Of these, the REDEEM trial [29] was the first reported randomized, double-blind, placebo-controlled trial for an intervention for 3 years in patients with PCA under AS. The REDEEM trial in patients with BPH used 0.5 mg of dutasteride, a 5α-reductase inhibitor that blocks the conversion of testosterone to dihydrotestosterone; a reduction in prostate volume and decreased PSA level were found. Of all prior reports, the REDEEM trial had the greatest number of enrolled patients. It was reported that 54 (38%) of 144 patients under AS in a dutasteride-treated group and 70 (48%) of 145 placebo-controlled patients under AS showed PCA progression (hazard ratio (HR): 0.62, 95% confidence interval (CI): 0.43–0.89, *p* < 0.01). Although not statistically significant compared to the placebo-controlled group, a slightly higher number of patients (35 or 24%) showed sexual AEs, including breast enlargement or tenderness, in the dutasteride group.

More recently, the ENACT trial [31], a phase II, randomized clinical trial, used 160 mg of enzalutamide (a second-generation antiandrogen agent) for 1 year, compared with AS alone. It was found that enzalutamide significantly reduced the cancer-progression risk by 46% compared with AS alone (HR: 0.54, 95% CI: 0.33–0.89, *p* < 0.05); the odds of a negative biopsy rate were 3.5 times higher. However, during the one-year treatment period, the number of AEs was higher in patients receiving enzalutamide (92%) compared with AS alone (54.9%). The most commonly reported AEs for enzalutamide were fatigue (55.4%), gynecomastia (36.6%), nipple pain (30.4%), breast dysfunction (25.9%), and sexual dysfunction (17.9%). Enrolled patients in our study had a slightly higher risk for disease progression than in prior AS studies; however, two patients had a negative prostate biopsy after 6 months of luteolin treatment and showed no AEs. In contrast to the aforementioned approved, commercially available drugs, supplemental flavonoid, including luteolin, may be appropriate interventional agents for patients under AS.

Recently, several studies have described mental stresses in patients with PCA receiving AS [34, 35]. In an analysis of 292 patients who received AS, Sypre et al. reported anxiety in 34.3% of cases in an AS-treated group versus 28.6% in a radical surgery group and 31.6% in a radiation therapy group (*p* = 0.400) [35]. Though not statistically significant, patients receiving AS tended to feel intense mental stress due to a fear of cancer progression. With this in mind, chemopreventive intervention in patients receiving AS is a reasonable and desirable approach, with luteolin an appropriate candidate in terms of the rarity of AEs. Furthermore, considering excellent results in terms of the preventive effect of an oral supplemental intake of luteolin in not only prostate carcinogenesis but also castration-resistant growth in our prior studies, a future phase II study of 50 mg luteolin for the prevention of progression in patients under AS may be justified by this pilot safety analysis.

miRNAs are small, noncoding RNAs (19–23 bases in length); most negatively regulate the expression of RNA transcripts in a sequence-dependent manner. Over the last decade, however, increasing evidence has shown that aberrant expression of miRNAs disrupts systematically controlled RNA networks in cancer cells. Such events are closely involved in cancer biology, including in progression and drug resistance [36, 37]. In a previous report, the luteolin treatment of a castration-resistant PCA cell-line, 22Rv1, led us to identify the upregulation of miRNA-29 and miRNA-30 families and the downregulation of AR and IGF1R as downstream targets of these miRNA families. In this current study, miRNA-29 and miRNA-30 were detected in serum and prostate gland samples of patients under AS. This led us to investigate whether luteolin can exert a preventive effect on progression by the downregulation of AR and NKX3.1 via the regulation of the aforementioned miRNAs. With regard to miRNA-29 and miRNA-30, several reports described the tumor suppressive effect of these in several cancers [38–41]. More specifically in PCA, Nishikawa et al. [38] reported that the restoration of miRNA-29 in castration-resistant PCA cells revealed a significant inhibition of cellular migration and invasion via direct regulation of the laminin *γ*1 gene. Kumar et al. [37] identified novel AR-regulating miRNAs by screening miRNA library. As a direct AR suppressive component, the inhibition of miRNA-30 enhanced AR expression and castration-resistant cell growth. We found a significant reduction of expression of miRNA-30 in metastatic castration-resistant PCA compared with healthy prostate tissue by RT-PCR analysis. However, no report exists describing the efficacy of serum miRNA-29 or miRNA-30 in PCA. Recently, biomarkers in serum and urine samples were analyzed with regard to the prediction of PCA progression in patients under AS [42, 43]. For instance, Gandellini et al. prospectively collected plasma samples at baseline from 386 patients under AS; a three-miRNA signature (miR-511-5p, miR-598-3p, and miR-199a-5p) was found to predict the grade reclassification of PCA in all patient cohorts [42]. Based on the biological mechanisms of luteolin in PCA, a biomarker analysis of such miRNAs might lead to the establishment of an appropriate treatment method for patients under AS; a future phase II study may contribute to this.

Our study had several limitations. Although the data originated from a novel treatment strategy, it was based on the analysis of a very limited sample size of five patients. Second, consecutive blood samples were compared with those from the same patients rather than controls. Therefore, it was impossible to perform a statistical analysis on the miRNA data. Third, the treatment period of this study was only six months.

In summary, in a single-arm, phase I study of luteolin in men under AS, we found that patients did not show AEs, suggesting luteolin treatment was safe. With regard to oncological outcomes, only two of the five patients studied showed disease progression as determined histologically, suggesting luteolin therapy was effective for PCA under AS. For more comprehensive data on the long-term effects and safety of luteolin, a long-term follow-up interventional study is required in future to support our study conclusions.

## 5. Conclusion

Daily oral supplementation with 50 mg luteolin for 6 months was found to be safe and may suppress PCA progression in patients under AS via the attenuation of expression of AR and NKX3.1. miR-29 and miR-30 induced by luteolin have important roles in reducing AR and NKX3.1 levels, resulting in inhibiting PCA progression. In addition, miR-29 and miR-30 may be novel therapeutic targets for PCA progression in clinical practice.

## Figures and Tables

**Table 1:** Patients' baseline characteristics.

Characteristics	Luteolin-treated group (*n* = 5)
Median age, year (range)	68 (61–79)
Initial PSA level, ng/mL (range)	7.2 (5.1–11.8)
ECOG-PS 0, *n* (%)	5 (100)
Maximum GG, *n* (%)	
1	4 (80)
2	1 (20)
Clinical T stage, *n* (%)	
T1c	2 (40)
T2a	3 (60)
Prostate cancer risk, *n* (%)	
Low	3 (60)
Intermediate	2 (40)
Type of biopsy at diagnosis, *n* (%)	
TRUS/mpMRI targeted	4 (80)
Non-US/mpMRI targeted	1 (20)
Type of repeated protocol biopsy, *n* (%)	
TRUS/mpMRI targeted	4 (80)
Non-US/mpMRI targeted	1 (20)

Abbreviations: ECOG-PS, Eastern Cooperative Oncology Group Performance Status; GG, grade group; mpMRI, multiparametric magnetic resonance imaging; PSA, prostate-specific antigen; TRUS, transrectal ultrasound.

## Data Availability

The data that support the findings of this study are available from the corresponding author upon reasonable request.

## Nomenclature

AEs: Adverse events
AR: Androgen receptor
AS: Active surveillance
BPH: Benign prostate hypertrophy
FFPE: Formalin-fixed paraffin-embedded
GG: Grade group
IGF-1R: Insulin-like growth factor-1 receptor
Luteolin: 3′, 4′, 5, 7-tetrahydroxy flavone
mpMRI: Multiparametric magnetic resonance imaging
PCA: Prostate cancer
PSA: Prostate-specific antigen
TRUS: Transrectal ultrasound
